# Computer-Based Assessment and Self-Report Measures of Executive Functions in High-Functioning Adults with Autism

**DOI:** 10.3390/brainsci12081069

**Published:** 2022-08-12

**Authors:** Ilinca Mihailescu, Lucia Emanuela Andrei, Alina Alexandra Frunza, Mirela Manea, Florina Rad

**Affiliations:** 1 Child and Adolescent Psychiatry Department, “Prof. Dr. Alexandru Obregia” Clinical Hospital of Psychiatry, 041914 Bucharest, Romania; 2 Child and Adolescent Psychiatry Department, Carol Davila University of Medicine and Pharmacy, 020021 Bucharest, Romania; 3 Psychiatry Department, Mina Minovici National Institute of Legal Medicine, 042122 Bucharest, Romania; 4 Department of Psychiatry and Psychology, Carol Davila University of Medicine and Pharmacy, 020021 Bucharest, Romania; 5 Department of Psychiatry, “Prof. Dr. Alexandru Obregia” Clinical Hospital of Psychiatry, 041914 Bucharest, Romania

**Keywords:** autism, executive functions, high-functioning autism

## Abstract

This study analyzes the profile of executive functions (EF) in high-functioning adults with autism (HFA), both in terms of performance on four computer-based tasks, as well as how these functions are perceived by the individuals through self-reporting measures. The study included 64 participants: 32 individuals with HFA, and 32 typically developing controls. Four CANTAB tasks were used (assessing spatial working memory, planning, visual memory, and inhibition), as well as a self-reported measure of executive functions (BDEFS) and a scale for the severity of autism symptoms (RAADS-R). The participants in the ASD group performed significantly lower than the control group on all four computer-based tasks, as measured by the total number of errors made (for the spatial working memory, visual memory, and inhibition tasks) and the number of problems solved at the first choice (for the planning task). No correlation was found in the ASD group between the severity of autism symptoms and the computer-based measures. These findings provide evidence that HFA adults may have various executive functioning impairments, and subsequent daily life problems, but these deficits do not necessarily correlate with the severity of core ASD symptoms.

## 1. Introduction

Autism spectrum disorder (ASD) is a complex neurodevelopmental disability involving persistent difficulties of social communication and restricted and repetitive interests and behaviors [1]. Although it is not an official clinical diagnosis, high-functioning autism (HFA) usually describes individuals with ASD but without an intellectual disability, who have independent living skills. Recent prevalence estimates show that 1 in 44 individuals have ASD, and among individuals identified with ASD, 35.2% also have an intellectual disability (IQ ≤ 70), 23.1% have an IQ between 71 and 85, and 41.7% have an IQ higher than 85 [2]. To date, ASD studies in adult populations have been considerably fewer than those carried out in pediatric populations, but existent community-based studies estimate a prevalence of ASD in adults between 0.9% and 2.2% [3,4,5,6].

Executive functions (EF) (e.g., inhibition, cognitive flexibility, planning, working memory, generativity) are a family of complex cognitive processes, coordinated by the frontal lobes that are needed to guide our thinking and behaviors toward certain goals. Executive functions help the human mind to make decisions, set goals, organize, plan, focus, learn etc., abilities which we need in all the activities of daily living.

Studies that attempt to analyze the profile of executive functions in ASD face several methodological obstacles, some arising from the high variability of this population and others being related to the difficulty of measuring the vast construct of executive functions in a more appropriate way. The performance of adults diagnosed with ASD without an intellectual disability on different EF tasks varies considerably, therefore there is no current consensus over the existence or absence of a specific EF deficit in adults with ASD. In a previous paper, we reviewed 48 studies on the performance of HFA in various EF tasks [7]. Taken all together, these studies confirm the lack of uniformity in the cognitive profile of the ASD population, either with the presence of a deficit in inhibition [8,9,10], planning [11], cognitive flexibility [12,13,14], working memory [13,15] and/or generativity [16,17], the absence of an impairment [18,19,20,21,22,23], or having mixed results [13,24,25,26,27,28]. In contrast to the abovementioned research, which used objective tasks for the assessment of different executive functions, the results obtained using self-report scales measuring these functions in daily life activities have proved to be more uniform, at least in terms of an overall higher score of ASD individuals, when compared to typically developing control groups [29,30,31]. 

The relationship between the core autism symptoms and the profile of executive functions in adults with ASD remains unclear. There are studies showing that the cognitive flexibility could not be correlated with the overall severity of autistic symptoms [14,32], and others revealing that flexibility can be correlated only with stereotyped behavior [24]. Another study reports that higher scores obtained in two different tests measuring autistic symptoms are associated with deficits in planning, problem-solving, inhibition and response initiation [33].

In the current study, we aim to analyze the profile of EF in HFA adults, both in terms of performance on four computer-based tasks, as well as how these functions are perceived by the individuals through self-report measures. Moreover, we aim to establish the relationship between these measurements and main deficits of ASD (i.e., social interaction, language, circumscribed interests, and sensory-motor problems).

## 2. Materials and Methods

### 2.1. Participants

The study included 64 participants, divided into two groups. The study group consists of 32 individuals (25 males and 7 females, aged 16 to 50 years old), with an autism spectrum disorder (ASD) diagnosis, according to DSM-5 criteria, and without an intellectual disability (IQ > 70). The selection of the participants was made from a pool of the former patients of the Child and Adolescent Psychiatry Department of the “Prof. Dr. Alexandru Obregia” Psychiatry Hospital (18 cases), and with the assistance of ASD-related associations from all over the country (14 cases). The ASD diagnosis was previously confirmed in 27 of the cases and newly diagnosed in five of the cases. The control group consisted of 32 typically developing individuals (21 males and 11 females, aged 16 to 56 years old). All the participants (both ASD and the control group) underwent a comprehensive psychiatric assessment performed by a psychiatrist, consisting of the medical and developmental history, the educational history, the mental status examination, a Diagnostic and Statistical Manual of Mental Disorders, Fifth Edition (DSM-5) diagnosis and the treatment history. The ASD diagnosis was confirmed in all 32 individuals in the study group. Participants who had visual, auditory or motor disorders of upper limbs, which could have influenced performance on computerized tasks or completing self-administered scales, were not included in the study, and neither were the individuals who had a psychotic disorder at the time of their inclusion in the study or a history of another neurodevelopmental disorder. All the individuals in the control group had no current diagnosis or history of a neurodevelopmental or psychiatric disorder.

Data collection took place between 2015 and 2018 and informed consent was obtained before inclusion (directly from the participants over 18 years old and from one of the parents for those participants between the ages of 16 and 18). The study was carried out in accordance with the ethical rules of research outlined in the 1995 Helsinki Declaration (revised at Edinburgh in 2000), and the study was approved by the Research Ethics Commission of the “Prof. Dr. Alexandru Obregia” Psychiatry Hospital in Bucharest.

### 2.2. Measures

The Ritvo Autism Asperger’s Diagnostic Scale-Revised [34] (RAADS-R) was used to evaluate the severity of ASD symptoms, the instrument consisting of 80 questions that investigate the current or past presence of specific symptoms, according to DSM IV-TR and ICD-10 criteria. If the participant had difficulties remembering the clinical situation in their childhood, then the help of a caregiver, usually a parent, was solicited. The items of the scale were divided into four subscales, each corresponding to a specific ASD domain, such as: social relatedness, circumscribed interests, language, and sensory-motor domain. The higher the total score resulting from adding the individual scores for each item, the greater the severity of the autistic spectrum symptomatology. The authors of the scale also set a cut-off value of 65 points, from which the ASD diagnosis can be formulated, with a sensitivity of 97% and a specificity of 100% [34].

The Barkley Deficits in Executive Functioning Scale [35] (BDEFS) was used to quantify the perception of the participants of the frequency with which they are confronted with certain difficulties deriving from the poor executive functioning. The 89 items are divided into five subscales: time management, organization and problem-solving, self-restraint, self-motivation, and self-regulation of emotions. To interpret the results according to the general population norms identified by the authors of the scale, a percentile rating is used, considering the participants’ gender and age category. Scores that exceed the 75th percentile are considered to reflect a clinically relevant executive dysfunction, with higher severity ratings corresponding to higher percentiles.

Four tests from the Cambridge Neuropsychological Test Automated Battery [36] (CANTAB) were performed to analyze executive functions. Versions of the tests with instructions in Romanian were used and the tests were carried out after the participant had spent some time familiarizing themselves with the device. All the participants in the control group and 30 participants from the initial ASD group performed the computerized tests, completing all four tasks described below (two of the participants chose to withdraw from the study before performing the CANTAB tests). In the statistical analysis of the data resulting from the CANTAB tests and the relationship between these results and the rest of the variables, data from 30 ASD individuals and 32 controls was included.

The Spatial Working Memory [36] (SWM) test is a task that evaluates spatial working memory and planning abilities, requiring remembering and manipulation of certain visual-spatial elements, as well as the use of an effective strategy. Several boxes appear on the screen, while a yellow token is hidden behind each of them. The number of tokens that needs to be found is the same as the number of boxes, but tokens can only be discovered one at a time, considering that a yellow token will never be found under a box that has already been used. The difficulty level increases gradually, as the number of boxes also increases. The test offers 18 parameters to be analyzed, out of which five are considered “key” outcome measures, which were also used in this study (total number of errors, errors in 4 boxes, errors in 6 boxes, errors in 8 boxes, and strategy score).

The One-Touch Stockings of Cambridge [36] (OTS) test is a variation of the Hanoi Tower test that evaluates planning ability. Two configurations appear on the screen, one at the top of the screen and one at the bottom, each containing three colored balls—pink, green and blue, placed in three “pockets”. The individual must mentally calculate the required number of movements of the balls needed to be made in the lower configuration, in order to obtain a configuration identical to the one in the upper display. The parameters used in this study are the total number of problems solved on first choice and the median latency to the first correct answer.

The Paired Association Learning [36] (PAL) test is a task that evaluates visual memory, while also containing a learning component. Several boxes appear on the screen and they open one by one, in a random order, one or more of them containing an abstract figure (that cannot be associated with objects or notions from the environment). The figures are then displayed one at a time, in the middle of the screen, and the participant must select the box in which they remember the figure that was initially displayed in. The key outcome measures included in this analysis are the total number of errors, the number of errors for each type of problem (2, 4, 6, and 8 figures) and the memory score (the number of times the subject chose the correct box at the first attempt).

The Multitasking Test [36] (MTT) evaluates inhibition and flexibility, it is a task that requires managing contradictory information and irrelevant stimuli inhibition. An arrow appears on the right or left half of the screen, with the arrowhead facing one of the directions—right or left. The participant is required to press one of the right or left buttons that are located at the bottom of the screen, depending on a word that appears on the upper screen, which can be “side”—meaning the half of the screen where the arrow is located or “direction”—meaning the direction in which the arrow is pointing. The test consists of three assignments, two of which show a single task and a multitasking part displaying both rules alternately in a random sequence, requiring flexibility in changing the rule as well as inhibiting the previous rule. The parameters used are the total number of errors, the number of congruent errors (those tasks where the direction of the arrow is the same as the part of the screen where the arrow was displayed), the number of incongruent errors (those tasks where the direction of the arrow is different from the side of the screen where the arrow was displayed), and the response latency.

### 2.3. Statistical Analysis

Statistical analysis was performed using SPSS version 17.0 (SPSS Inc. Released 2008. SPSS Statistics for Windows, Version 17.0. Chicago, IL, USA: SPSS Inc.) and both descriptive and inferential statistics were used. Continuous data were summarized using either the median or the mean and standard deviation, depending on the distribution of data. Mann–Whitney U, independent *t*-tests, or Chi-square tests were used to analyze group differences in RAADS-R scores, BDEFS scores, and computer-based measures. For the Spatial working memory test and the Paired Association Learning test, mixed ANOVA models were used to determine whether there is an interaction between the difficulty level (within-subjects factor) and the group (ASD or control, between-subjects factor) on the total numbers of errors made by the participants (dependent variable). Spearman’s correlations were further performed in the ASD group, in order to determine the association between the severity of different ASD symptoms (RAADS-R scores) and the measures of executive dysfunction (BDEFS scores and computer-based tests scores). Statistical significance was set at *p* < 0.01. Effect sizes were also reported (Cohen’s *d* or η^2^) to determine the magnitude of the obtained differences.

## 3. Results

The results obtained on all the five subscales of the BDEFS instrument show the existence of significant differences between the two groups, the scores on Time management, Organization and problem-solving, Self-restraint, Self-motivation and Self-regulation of emotions scales being higher in the ASD group (Table 1). According to the effect size calculations, differences are important (η^2^ > 0.13), the largest one being identified in the Organization and problem-solving scale. According to the normative percentile values provided by the authors of BDEFS scale, the results were divided into the following categories: normal range (1st–75th percentiles), subclinical range (percentiles 76th–92nd), mild deficit (93rd–95th percentiles), moderate deficit (percentiles 96th–98th) and severe deficit (≥99th percentile). Thus, the percentages of participants in the ASD group, reporting the presence of any deficits (≥93rd percentile), are the following: 35.4% on the BDEFS Time management scale (compared to 3.1% in the control group, χ^2^(4) = 13.41, *p =* 0.00), 54.7% on the BDEFS Organization and problem-solving scale (compared to 6.2% in the control group, χ^2^(4) = 23.48, *p =* 0.00), 41.8% on the BDEFS Self-restraint scale (compared to 9.3% in the control group, χ^2^(4) = 10.09, *p =* 0.02), 64.4% on the BDEFS Self-motivation scale (compared to 21.8% in the control group, χ^2^(4) = 14.66, *p =* 0.00), 38.5% on the BDEFS Self-regulation of emotions scale (compared to 12.4% in the control group, χ^2^(4) = 16.38, *p =* 0.00) and 54.7% on BDEFS Total score (compared to 6.2% in the control group, χ^2^(4) = 23.05, *p =* 0.00).

Table 2 shows the results of ASD and control groups on the computer-based EF tests. The number of errors on the Spatial Working Memory (SWM) task is significantly higher in the ASD group, both at each level of difficulty (4, 6, and 8 boxes), as well as in the total number of errors (Table 2). The interaction between the group and the SWM difficulty level (2 × 3 model) is significant, F(2, 118) = 5.42, *p* = 0.01, ηp^2^ = 0.08, as the difference between the two groups increases with a higher level of difficulty. The results obtained on the One-Touch Stockings of Cambridge task show a lower number of problems solved on first choice in the ASD group, but without any difference in response latency (Table 2). The total number of errors on Paired Association Learning (PAL) task is greater in the ASD group and the memory score is significantly lower (Table 2). Significant difference between the two groups was found in each of the PAL tasks, as participants in the ASD group made more errors on the 4-, 6-, and 8- boxes tasks, apart from the easiest one (2 boxes), where no significant difference was observed. The interaction between the group and the PAL difficulty level (2 × 4 model) is significant, F(3, 174) = 22.76, *p* = 0.00, ηp^2^ = 0.28, as the difference between the two groups increases with a higher level of difficulty. The results obtained on Multitasking Test (MTT) show a higher total number of errors in the ASD group (Table 2), with more errors both in congruent tasks (U = 301.1, *p* = 0.01, η^2^ = 0.26) and incongruent tasks (U = 175.5, *p* = 0.00, η^2^ = 0.08).

The analysis between the severity of ASD symptoms (RAADS-R scores) and self-reported measures of EF (BDEFS scores) in the ASD group, shows moderate significant correlations between RAADS-R sensory-motor domain and several BDEFS subscales (Organization and problem-solving, r = 0.43, *p* = 0.01; Self-motivation, r = 0.37, *p* = 0.03; Self-regulation of emotions, r = 0.45, *p* = 0.01), as well as the BDEFS total score (r = 46, *p* = 0.00) (Table 3). No correlation has been found in the ASD group, between any of the RAADS-R scores and the results of the four computer-based tasks (Table 3).

## 4. Discussion

In this study, we investigated the performance of a group of adult individuals with high-functioning autism, on four computer-based tests measuring spatial working memory, planning, visual memory, and inhibition, but we also measured the self-reported difficulties deriving from the poor executive functioning.

One of the main results of this research, derived from the computer-based assessments, is that the participants with ASD performed significantly lower than their typically developing counterparts on spatial working memory, planning, visual memory, and inhibition tasks. These findings are in line with those reported by other studies investigating these EF with various types of measurements [8,9,10,11,13,15], but in opposition to those failing to find any deficit [18,19,20,21,22,23]. The very high variability of the results can be attributed to a potential phenotypic heterogeneity of the cognitive profile among people with ASD, but also to notable differences between the methodologies used, especially regarding the instruments chosen to measure executive functions. Another problem raised by some authors conducting neuro-psychological research is whether these various measurements used in EF studies have a proper ecological validity, in terms of how test performance can be generalized to real-life situations. Regarding this issue, one study [27] investigated inhibition in adults with high-functioning autism, using four Stroop tasks: a classic paper version, a computerized version, and two Stroop tasks using virtual reality techniques-with external, visual and voice distractors and without any external distractors. The study reports a lack of significant differences between adults with ASD and neuro-typical controls on the classic, computerized, and virtual reality without external distractors versions of the Stroop test, but with the presence of deficits in individuals with ASD on the virtual reality with external distractors test [27]. The authors conclude that the inhibitory response is possibly affected in adults with ASD but, as with other executive functions, regular laboratory measurements do not reflect the complexity of the real world, presenting an important problem of ecological validity [27].

The participants with ASD in our study also report significantly greater problems with time management, organization and problem-solving, self-restraint, self-motivation, and self-regulation of emotions (BDEFS domains), compared to typically developing individuals. Comparing our findings with normative data, provided by the authors of the scale, following the evaluation of 1249 typically developing individuals from the general population, the most frequent clinical deficits in our ASD group are found in the organization and problem-solving and self-motivation domains. The use of tools for the subjective assessment of executive dysfunction is increasingly common, given their superior ecological validity. The results of this study are consistent with those obtained by other studies, using the Behavioral Rating Inventory of Executive Function [37] (BRIEF) questionnaire, with higher overall scores reported by ASD individuals compared to those reported by typically developing subjects [23,29,38]. Among the most affected domains identified by individuals with ASD in these studies are organization/planning, flexibility, and emotional control [23,29,38].

In this study, no significant correlation has been found between the performance on the computer-based tasks and the severity of ASD symptoms. A moderate correlation was identified between the self-reported EF deficits (BDEFS Total score) and the severity of sensory-motor problems reported by ASD participants, but not with the other ASD symptoms (i.e., social relatedness, circumscribed interests, and language problems). These findings can be explained by the way the individuals with ASD perceive that different ASD symptoms interfere with their daily skills of organization, planning, time management, emotional regulation, etc. Sensory difficulties (e.g., annoying noises, the way some objects or items of clothing feel, food consistency, etc.) may cause more difficulty in activities that require good executive functioning.

This study also faces some limitations. The most important is the one related to the absence of the exact IQ level of each participant with the impossibility of matching the groups in terms of cognitive performance, which could have impacted the group differences on computer-based tasks. Thus, although the two groups were similar in age, gender, and educational level and although neither participant had a diagnosis of intellectual disability following an IQ exam taken in the past, we did not have access to the exact IQ level.

## 5. Conclusions

Individuals with high-functioning autism report significant difficulties deriving from the poor executive functioning, difficulties related to time management, organisation and problem-solving, self-motivation, self-restraint, and self-regulation of emotions. Our findings also suggest a potentially more impaired performance in executive function tasks (spatial working memory, visual memory, planning abilities and inhibition), when compared to typically developing individuals. Almost all of the abovementioned self-reported difficulties and EF performance do not correlate with the severity of ASD symptoms in HFA, except for the sensory difficulties of these individuals that have been associated with the perceived level of EF dysfunction in daily activities. The heterogeneity of the results from similar studies, which either indicates the presence or the absence of EF deficits, could be explained by an unequal profile of these functions in ASD, a profile that should be determined and subsequently used in clinical practice. In addition, in order to have a more comprehensive outlook, we believe it is necessary to add to this cognitive profile the impact that it has on daily life activities, using tools with adequate ecological validity. It is our belief that this potentially comprehensive executive functioning profile could be used to design individualized therapeutic interventions.

## Figures and Tables

**Table 1:** Demographic and clinical characteristics.

	ASD (n = 32) (Mean ± SD/mdn)	Control (n = 32) (Mean ± SD/mdn)	Statistics	*p*	Effect Size
Age	25.03 ± 9.6	25.72 ± 9.7	U = 474.5	0.61	
Gender					
Male	25 (78.1%)	21 (65.7%)	χ^2^ = 1.23	0.70	
Female	7 (21.9%)	11 (34.3%)			
Last level of education					
8–12 grades	19 (59.3%)	18 (56.2%)	χ^2^ = 0.06	0.80	
University	13 (40.6%)	14 (43.8%)			
RAADS-R					
Total score	119.00 ± 31.26	30.87 ± 16.78	t = 13.99	0.00	*d* = 3.49
Social relatedness	60.5	11.5	U = 3.50	0.00	*η*^2^ = 0.72
Circumscribed interests	28.0	9.0	U = 60.0	0.00	*η*^2^ = 0.57
Language	6.0	0.0	U = 95.0	0.00	*η*^2^ = 0.48
Sensory-motor	25.5	7.0	U = 64.5	0.00	*η*^2^ = 0.55
BDEFS					
Total score	204.97 ± 59.25	140.00 ± 33.09	t = 5.35	0.00	*d* = 1.34
Time management	49.0	33.0	U = 234.0	0.00	*η*^2^ = 0.20
Organization and problem-solving	55.0	32.0	U = 140.5	0.00	*η*^2^ = 0.37
Self-restraint	38.0	28.5	U = 238.5	0.00	*η*^2^ = 0.19
Self-motivation	28.0	17.5	U = 222.0	0.00	*η*^2^ = 0.22
Self-regulation of emotions	31.0	20.0	U = 223.5	0.00	*η*^2^ = 0.22

ASD = autism spectrum disorder; BDEFS = Barkley Deficits in Executive Functioning Scale; mdn = median; RAADS-R = Ritvo Autism Asperger Diagnostic Scale–Revised; SD = standard deviation.

**Table 2:** Performance on the computer-based EF tests (key outcome measures).

	ASD (n = 30) (Mean ± SD/mdn)	Control (n = 32) (Mean ± SD/mdn)	Statistics (U)	*p*	Effect Size (η^2^)
*Spatial Working Memory (SWM)*					
Between Errors–Total	16.1 ± 8.7	8.9 ± 9.6	282.5	0.00	0.12
Strategy score	9.1 ± 2.0	6.4 ± 251.5	251.5	0.00	0.16
*One-Touch Stockings of Cambridge (OTS)*					
Problems solved on first choice–Total	8.6 ± 4.0	12.4 ± 1.5	182.0	0.00	0.28
Median Latency to first choice–Total	14.17	11.25	407.0	0.53	0.00
*Paired Association Learning (PAL)*					
Total errors-adjusted	18.0	4.0	114.0	0.00	0.41
Memory score	11.0	17.0	157.0	0.00	0.31
*Multitasking Test (MTT)*					
Total errors	8.0	2.0	180.5	0.00	0.26
Reaction latency	627.5	617.5	385.5	0.34	0.01

ASD = autism spectrum disorder; mdn = median; SD = standard deviation.

**Table 3:** Correlations between the RAADS-R scores and the executive functions measures in the ASD group (Spearman’s correlation coefficients).

	RAADS-R Total Score	Social Relatedness	Circumscribed Interests	Language	Sensory-Motor
*BDEFS*					
Total score	0.34	0.24	0.26	0.09	0.46 **
Time management	0.119	0.32	0.22	0.11	0.34
Organization and problem-solving	0.31	0.19	0.26	0.07	0.43 *
Self-restraint	0.20	0.13	0.19	−0.06	0.35
Self-motivation	0.23	0.11	0.21	−0.06	0.38 *
Self-regulation of emotions	0.30	0.14	0.23	0.16	0.45 *
*Spatial Working Memory*					
Total errors	0.06	−0.06	0.15	0.18	0.03
*One-Touch Stockings of Cambridge*					
Problems Solved on first choice	0.08	0.12	0.06	0.01	−0.04
*Paired Association Learning*					
Total Errors	−0.02	0.04	−0.08	0.04	0.01
*Multitasking Test*					
Total Errors	0.03	0.01	−0.09	0.04	0.21

* *p* < 0.05; ** *p* < 0.01. RAADS-R = Ritvo Autism Asperger Diagnostic Scale–Revised; BDEFS = Barkley Deficits in Executive Functioning Scale.

## Data Availability

The data are not publicly available. The datasets used and analyzed are available from the corresponding author on reasonable request.

## References

[1] American Psychiatric Association (2013). Diagnostic and Statistical Manual of Mental Disorders (DSM-5).

[2] Maenner M.J., Shaw K.A., Bakian A.V., Bilder D.A., Durkin M.S., Esler A., Furnier S.M., Hallas L., Hall-Lande J., Hudson A. (2021). Prevalence and Characteristics of Autism Spectrum Disorder Among Children Aged 8 Years—Autism and Developmental Disabilities Monitoring Network, 11 Sites, United States, 2018. MMWR Surveill. Summ..

[3] Brugha T., Cooper S.A., McManus S., Purdon S., Smith J., Scott F.J., Spiers N., Tyrer F. (2012). Estimating the Prevalence of Autism Spectrum Conditions in Adults: Extending the 2007 Adult Psychiatric Morbidity Survey.

[4] Kočovská E., Biskupstø R., Gillberg I.C., Ellefsen A., Kampmann H., Stórá T., Billstedt E., Gillberg C. (2012). The Rising Prevalence of Autism: A Prospective Longitudinal Study in the Faroe Islands. J. Autism Dev. Disord..

[5] Idring S., Lundberg M., Sturm H., Dalman C., Gumpert C.H., Rai D., Lee B., Magnusson C. (2015). Changes in Prevalence of Autism Spectrum Disorders in 2001–2011: Findings from the Stockholm Youth Cohort. J. Autism Dev. Disord..

[6] Dietz P.M., Rose C.E., McArthur D., Maenner M. (2020). National and State Estimates of Adults with Autism Spectrum Disorder. J. Autism Dev. Disord..

[7] Mihailescu I., Frunza A.A., Andrei E.L., Rad F., Dobrescu I., Manea M. (2018). A review of performance-based executive function tasks in adults with autism and normal intelligence. Rom. J. Psychiatry.

[8] Wilson C.E., Happé F., Wheelwright S.J., Ecker C., Lombardo M.V., Johnston P., Daly E., Murphy C.M., Spain D., Lai M.-C. (2014). The Neuropsychology of Male Adults With High-Functioning Autism or Asperger Syndrome. Autism Res..

[9] Johnston K., Madden A.K., Bramham J., Russell A.J. (2011). Response Inhibition in Adults with Autism Spectrum Disorder Compared to Attention Deficit/Hyperactivity Disorder. J. Autism Dev. Disord..

[10] Luna B., Doll S.K., Hegedus S.J., Minshew N.J., Sweeney J.A. (2007). Maturation of Executive Function in Autism. Biol. Psychiatry.

[11] Lopez B.R., Lincoln A.J., Ozonoff S., Lai Z. (2005). Examining the Relationship between Executive Functions and Restricted, Repetitive Symptoms of Autistic Disorder. J. Autism Dev. Disord..

[12] Braden B.B., Smith C.J., Thompson A., Glaspy T.K., Wood E., Vatsa D., Abbott A.E., McGee S.C., Baxter L.C. (2017). Executive function and functional and structural brain differences in middle-age adults with autism spectrum disorder. Autism Res..

[13] Kiep M., Spek A.A. (2017). Executive functioning in men and women with an autism spectrum disorder. Autism Res..

[14] Yasuda Y., Hashimoto R., Ohi K., Yamamori H., Fujimoto M., Umeda-Yano S., Fujino H., Takeda M. (2014). Cognitive inflexibility in Japanese adolescents and adults with autism spectrum disorders. World J. Psychiatry.

[15] Bodner K.E., Beversdorf D., Saklayen S.S., Christ S.E. (2012). Noradrenergic Moderation of Working Memory Impairments in Adults with Autism Spectrum Disorder. J. Int. Neuropsychol. Soc..

[16] Kasirer A., Mashal N. (2014). Verbal creativity in autism: Comprehension and generation of metaphoric language in high-functioning autism spectrum disorder and typical development. Front. Hum. Neurosci..

[17] Kleinhans N.M., Müller R.-A., Cohen D.N., Courchesne E. (2008). Atypical functional lateralization of language in autism spectrum disorders. Brain Res..

[18] Ambery F.Z., Russell A.J., Perry K., Morris R., Murphy D. (2006). Neuropsychological functioning in adults with Asperger syndrome. Autism.

[19] Shafritz K.M., Bregman J.D., Ikuta T., Szeszko P.R. (2015). Neural system mediating decision-making and response inhibition for social and non social stimuli in autism. Prog. Neuro-Psychopharmacol. Biol. Psychiatry.

[20] Towgood K.J., Meuwese J.D., Gilbert S.J., Turner M.S., Burgess P.W. (2009). Advantages of the multiple case series approach to the study of cognitive deficits in autism spectrum disorder. Neuropsychologia.

[21] Losh M., Adolphs R., Poe M.D., Couture S., Penn D., Baranek G., Piven J. (2009). Neuropsychological Profile of Autism and the Broad Autism Phenotype. Arch. Gen. Psychiatry.

[22] Baez S., Rattazzi A., Gonzalez-Gadea M.L., Torralva T., Vigliecca N.S., Decety J., Manes F., Ibanez A. (2012). Integrating intention and context: Assessing social cognition in adults with Asperger syndrome. Front. Hum. Neurosci..

[23] Davids R.C.D., Groen Y., Berg I.J., Tucha O.M., van Balkom I.D.C. (2016). Executive Functions in Older Adults With Autism Spectrum Disorder: Objective Performance and Subjective Complaints. J. Autism Dev. Disord..

[24] Sachse M., Schlitt S., Hainz D., Ciaramidaro A., Schirman S., Walter H., Poustka F., Bölte S., Freitag C.M. (2013). Executive and Visuo-motor Function in Adolescents and Adults with Autism Spectrum Disorder. J. Autism Dev. Disord..

[25] Williams D.L., Goldstein G., Carpenter P.A., Minshew N.J. (2005). Verbal and Spatial Working Memory in Autism. J. Autism Dev. Disord..

[26] Williams D.L., Goldstein G., Minshew N.J. (2005). Impaired memory for faces and social scenes in autism: Clinical implications of memory dysfunction. Arch. Clin. Neuropsychol..

[27] Parsons T.D., Carlew A.R. (2016). Bimodal Virtual Reality Stroop for Assessing Distractor Inhibition in Autism Spectrum Disorders. J. Autism Dev. Disord..

[28] Lever A.G., Werkle-Bergner M., Brandmaier A.M., Ridderinkhof K.R., Geurts H.M. (2015). Atypical working memory decline across the adult lifespan in autism spectrum disorder?. J. Abnorm. Psychol..

[29] Ikuta T., Shafritz K.M., Bregman J., Peters B.D., Gruner P., Malhotra A.K., Szeszko P.R. (2014). Abnormal cingulum bundle development in autism: A probabilistic tractography study. Psychiatry Res. Neuroimaging.

[30] Wallace G.L., Kenworthy L., Pugliese C.E., Popal H., White E.I., Brodsky E., Martin A. (2016). Real-World Executive Functions in Adults with Autism Spectrum Disorder: Profiles of Impairment and Associations with Adaptive Functioning and Co-morbid Anxiety and Depression. J. Autism Dev. Disord..

[31] Cederlund M., Hagberg B., Gillberg C. (2010). Asperger syndrome in adolescent and young adult males. Interview, self—and parent assessment of social, emotional, and cognitive problems. Res. Dev. Disabil..

[32] Sumiyoshi C., Kawakubo Y., Suga M., Sumiyoshi T., Kasai K. (2011). Impaired ability to organize information in individuals with autism spectrum disorders and their siblings. Neurosci. Res..

[33] Hill E.L., Bird C.M. (2006). Executive processes in Asperger syndrome: Patterns of performance in a multiple case series. Neuropsychologia.

[34] Ritvo R.A., Ritvo E.R., Guthrie D., Ritvo M.J., Hufnagel D.H., McMahon W., Tonge B., Mataix-Cols D., Jassi A., Attwood T. (2011). The Ritvo Autism Asperger Diagnostic Scale-Revised (RAADS-R): A Scale to Assist the Diagnosis of Autism Spectrum Disorder in Adults: An International Validation Study. J. Autism Dev. Disord..

[35] Barkley R.A. (2011). Barkley Deficits in Executive Functioning Scale (BDEFS).

[36] CANTAB^®^ [Cognitive Assessment Software] Cambridge Cognition (2019). All Rights Reserved. www.cantab.com.

[37] Gioia G.A., Isquith P.K., Guy S.C., Kenworthy L. (2000). Test Review—Behavior Rating Inventory of Executive Function. Child Neuropsychol..

[38] Dijkhuis R.R., Ziermans T., van Rijn S., Staal W.G., Swaab H. (2017). Self-regulation and quality of life in high-functioning young adults with autism. Autism.

